# Riedel's thyroiditis - a case report with genes' expression studies

**DOI:** 10.1186/1756-6614-5-2

**Published:** 2012-03-07

**Authors:** Katarzyna Wojciechowska-Durczyńska, Adam Durczyński, Stanisław Sporny, Janusz Strzelczyk, Andrzej Lewiński

**Affiliations:** 1 Department of Endocrinology and Metabolic Diseases, Polish Mother's Memorial Hospital Research Institute, Medical University of Lodz, Rzgowska St. No. 281/289, 93-338 Lodz, Poland; 2 Department of General and Transplant Surgery, Medical University of Lodz, Barlicki University Hospital, Lodz, Poland; 3 Department of Dental Pathology, Medical University of Lodz, The Clinical-Didactic Centre, Lodz, Poland

**Keywords:** Riedel's thyroiditis, Genes' expression, Real-time PCR

## Abstract

**Background:**

Genetic background of Riedel's thyroiditis remains unknown. Herein, we describe our results of studies on genes expression levels in Riedel's thyroiditis.

**Case report and genetic findings:**

We report the case of 48-year old woman with Riedel's thyroiditis who has presented unusual course of disease with non-specific cervical discomfort, though as with no pain and/or no compression symptoms. After surgery, thyroid specimens were quantitatively evaluated, regarding *PIK3CA, PIK3CD, PIK3CG, Tg, TGFB1, THRB, COL1, CDKN1C, CDH3 *and *CACNA2D2 *genes expression levels, by real-time PCR in the ABI PRISM^® ^7500 Sequence Detection System. Out of 10 above genes, in 2 cases the expression was higher than in respective Controls of unchanged thyroid tissue. In the remaining 8 cases, expression in question became comparable or lower as in Controls.

**Discussion:**

The association between increased expression levels of *PIK3CA *and *CDH3 *genes and Riedel's thyroiditis is not well-defined. However, the increased expression of *PIK3CA *and *CDH3 *genes in our case report and in previous studies of other authors on various malignancies may suggest possible molecular relation between Riedel's thyroiditis and certain neoplastic processes, the relation of which requires further genetic evaluation. It is to be stressed that gene expression studies in Riedel's thyroiditis are difficult to perform, mainly due to fibrosis, resulting in scarce thyroid specimens and - in consequence - small amount of genetic material.

## Background

Riedel's thyroiditis is a rare fibrosclerotic infiltrative thyroid disorder of unclear etiology, during which normal thyroid tissues are replaced by fibrous tissue, often expanding outside the thyroid capsule. The diagnosis can be difficult to establish prior to surgical removal of the thyroid and to subsequent histopathological examination of the resected gland [1-3]. Reports on genetic findings in Riedel's thyroiditis are scarce in medical literature. Herein, we describe our results of studies on genes expression levels in Riedel's thyroiditis.

## Case report

**Figure 1:**
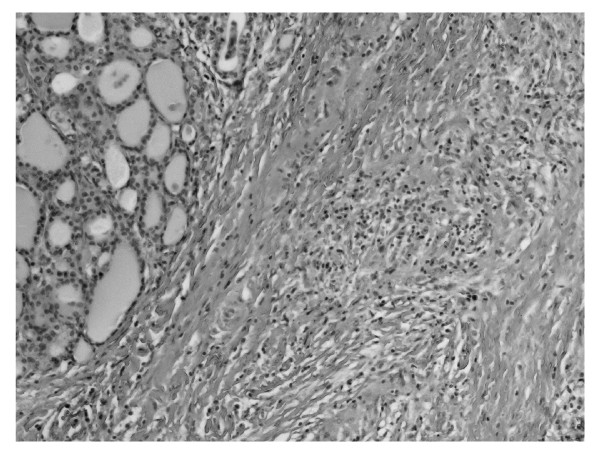
**Thyroid parenchyma peripherally to dense connective tissue hyperplasia**. HE 320 ×.

**Figure 2:**
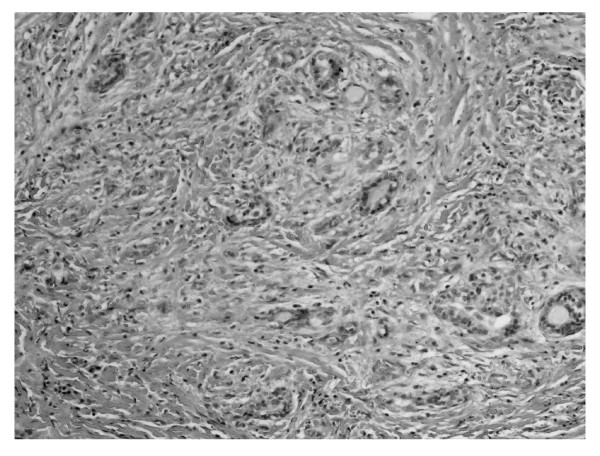
**Thyroid parenchyma remnant among dense connective tissue**. HE 320 ×.

**Figure 3:**
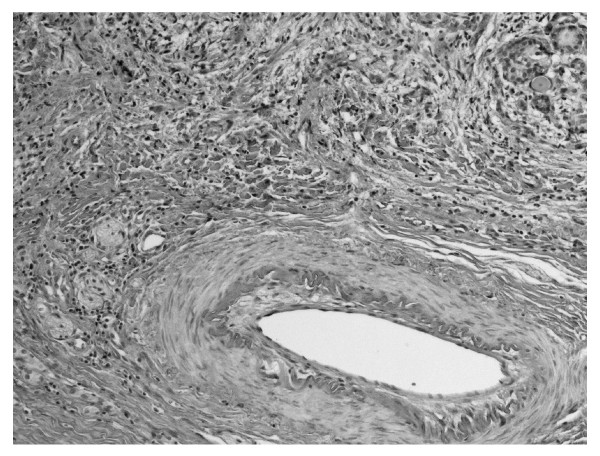
**Arterial vessel among fibrosing thyroid parenchyma**. HE 320 ×.

**Figure 4:**
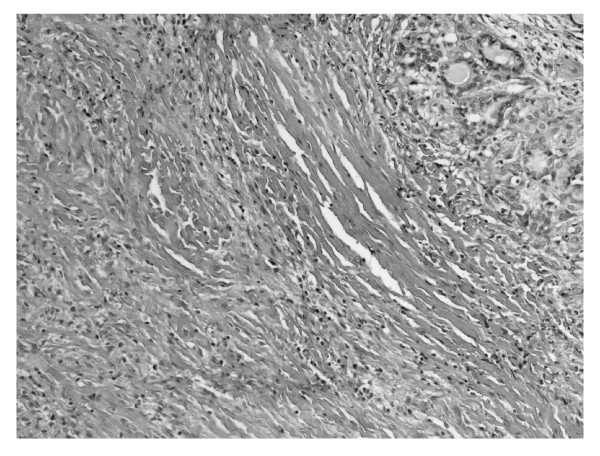
**Thyroid parenchyma peripherally to dense connective tissue hyperplasia**. HE 320 ×.

We report the case of 48-year old women, diagnosed originally as a goiter with eminently increased consistency on palpation, presented with non-specific cervical discomfort, though with no pain and/or no compression symptoms. Clinically, the thyroid was enlarged, painless, however very firm. Serum thyrotropin (TSH) and free thyroid hormones (FT3 and FT4) concentrations were in normal ranges. Prior to surgery, the result of chest X-ray examination showed no pathological changes and all biochemical laboratory tests were normal. Ultrasound examination of the thyroid gland revealed few normoechoic foci in both lobes. Based on benign cytology of the nodules (normal thyroid follicular cells and colloid - Bethesda II) and the absence of compression symptoms, a subtotal thyroidectomy was performed. During surgery, a stony hard, white thyroid was disclosed. No complications after thyroid surgery were observed. Histopathological findings led to the diagnosis of Riedel's thyroiditis, based on disappearance of thyroid follicles, which were replaced by the fibrous tissue and lymphocytes infiltrations, and features of focal endoarteritis (Figures 1, 2, 3, 4). Postoperatively, the patient was treated with levothyroxine, 100 μg per day. At 4-months follow up, results TSH, FT3, FT4, PTH, calcium-phosphorus homeostasis and autoantibodies against thyroid peroxidase and TSH receptor were in normal ranges; autoantibodies against thyroglobulin (anti-Tg) were elevated. Ultrasound examination of the neck did not showed features of disease recurrence.

## Genetic findings

After surgery, following conventional histopathological examination, thyroid specimen, received from patient with Riedel's thyroiditis, was quantitatively evaluated regarding *PIK3CA, PIK3CD*, *PIK3CG, Tg, TGFB1, THRB, COL1, CDKN1C, CDH3 and CACNA2D2 *genes expression levels by real-time PCR in the ABI PRISM ^®^7500 Sequence Detection System. Macroscopically unchanged thyroid tissue, surgically removed from the patient with non-toxic nodular goitre (NTG), served as a control for *real-time *PCR experiment.

Total RNA from tissue was extracted by use of an RNeasy Midi Kit (Qiagen, Hilden, Germany) based on modified Chomczyński and Sacchi's according to manufacturers' recommendations. The purity of total RNA was assessed by NanoDrop^® ^ND-100 spectrophotometr and next the RNA was used in the first strand cDNA synthesis with TaqMan^® ^Reverse Transcripton Reagents (Applied Biosystem) according to maufacturers' instruction. Real-time PCR was performed by using *Taq*Man^® ^Universal PCR Master Mix (Applied Biosystem) and *Taq*Man^® ^Gene Expression Assays probe and primer mix (Applied Biosystem) according to the manufacturer' specification. Thermal cycler conditions were as follows: hold for 10 min. at 95°C, follow by two-step PCR for 50 cycles of 95°C for 15 s followed by 60°C for 1 min. Amplification reactions, in triplicate for each sample, were performed and the results were normalized to the *ACTB *gene expression level.

An analysis of relative gene expression data was performed, using the 2^-ΔΔCT ^method on an ABI PRISM^® ^7500 Sequence Detection System Software. The fold change in studied gene expression, normalised to endogenous control, was calculated using: RQ = 2^-ΔΔCT^.

**Figure 5:**
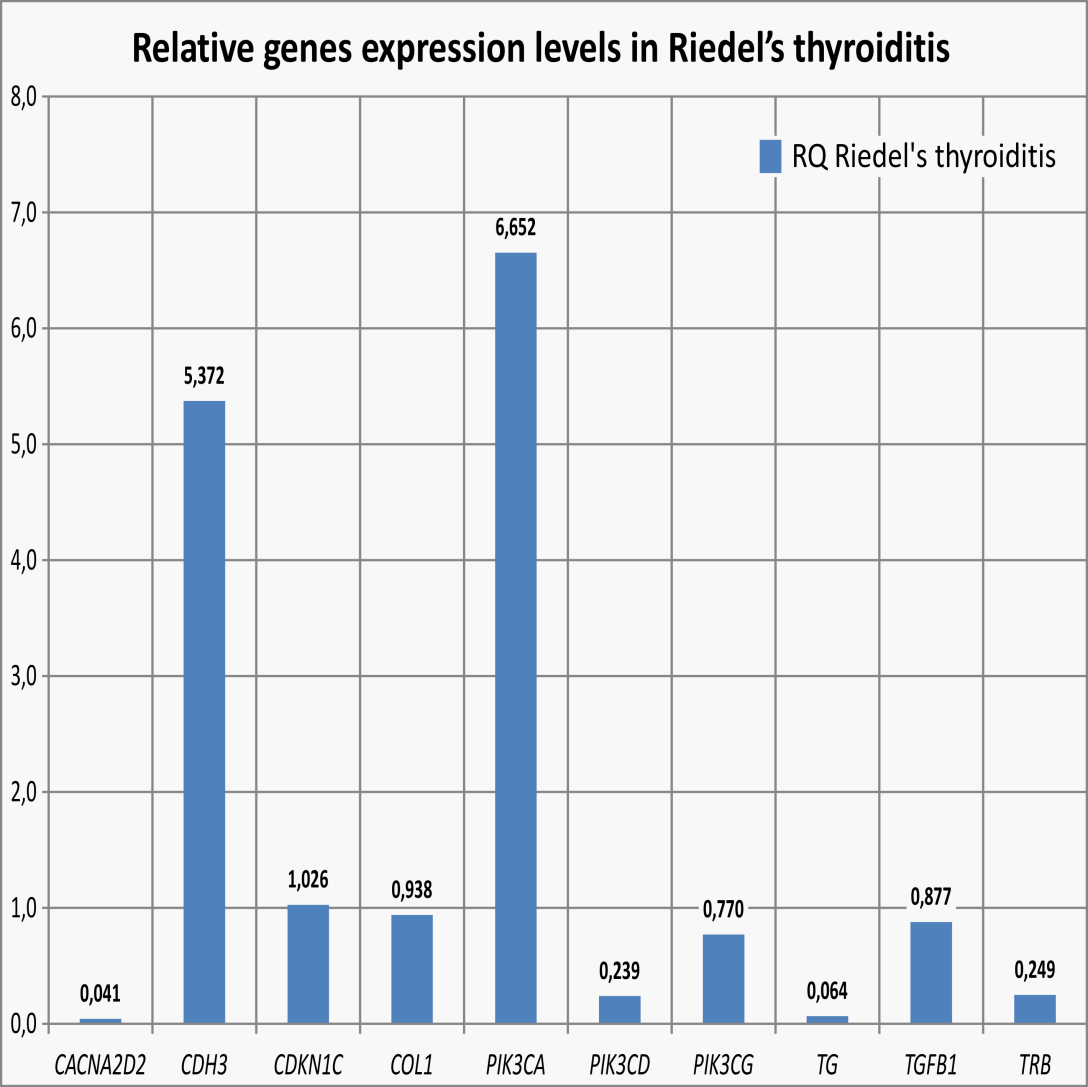
**Real-time PCR relative quantification (RQ) of studied genes levels in Riedel's thyroiditis**. Bars represents quantification values normalized to an endogenous control (β-actin) and relative to a calibrator (control sample, RQ = 1).

Out of 10 above genes, in 2 cases [*PIK3CA *- responsible for coding alpha catalytic subunit of class I PI3K (phosphoinositide 3-kinase) and *CDH3 *- responsible for coding P-cadherin] the expression was higher than in respective Controls of unchanged thyroid tissue. In the remaining 8 cases, expression in question became comparable or lower as in Controls (Figure 5).

## Discussion

Riedel's throiditis is a rare disease that most commonly occurs in females in their fifties and usually demonstrates as a goitre without clinical evidence of thyroid nodules. On palpation, thyroid demonstrates firm or stony consistency ("iron-hard thyroiditis"). Symptoms of regional compression might progressively appear. However, the clinical course of this disease is mostly variable and may include cases with no thyroid-related symptoms, as we have noted in our patient [3]. Before surgery, Riedel's thyroiditis is often difficult to differentiate from aggressive types of thyroid cancers (i.e., undifferentiated thyroid carcinoma), nevertheless, a long-term follow-up shows no disease-specific mortality [4]. Likewise in presented patient, in vast majority of cases, only histopathological, postoperative examination leads to diagnosis of Riedel's thyroiditis, due to no specific symptoms. Cytological evaluation is also of limited use in Riedel's thyroiditis, due to firm thyroid tissue consistency and scanty amount of cellular material thus often non-diagnostic thyroid specimens [5]. According to our opinion, in the case described by us, unusual course of the Riedel's thyroiditis with the absence of compressive symptoms may be related to early-stage of the disease.

A number of hypotheses have been proposed regarding etiology and pathogenesis of Riedel's thyroiditis. The disease may be associated with subacute and chronic thyroiditis, thus some authors proposed the, so called, "intrathyroidal hypothesis". According to that hypothesis, the entity may represent the late stage (i.e., the progression to fibrotic stage) of chronic inflammatory disorder [6-8]. On the contrary, the "pharmacological hypothesis" suggests that some particular medications may trigger Riedel's thyroiditis development [8]. Although not proven, inherited susceptibility and development was hypothesized, as well [8]. Nowadays, the "systemic autoimmune hypothesis" seems to have the most support and it basically views Riedel's thyroiditis as a manifestation of disordered fibroplastic proliferation because of hypersensivity reaction and consequent release of stimulating growth factors [9]. This theory considers a fibroblast not only as a final cell responsible for fibrosis, but also as a main target for well-defined systemic autoimmune attack [8]. In our case, the "systemic autoimmune hypothesis" of Riedel's thyroiditis is the most plausible, it is supported by laboratory findings of autoimmune thyroid disease (anti-Tg), which have persisted after surgery.

PI3K/Akt (phosphoinositide 3-kinase/serine-threonine protein kinase) pathway participates in cellular signaling in response to various growth factors including FGF (fibroblast growth factor). Activating mutations and amplification of *PIK3CA *gene have been associated with enhancing PIK3CA kinase activity and Akt phosphorylation. Increased PI3K/Akt expression has been observed in both Hashimoto thyroiditis and well-differentiated thyroid cancer [10,11]. Still, little is known about the precise role of *PIK3CA *in fibrosclerotic disorders, however, it has been proved that hepatic Akt expression correlated with advanced liver fibrosis in chronic hepatitis C patient [12].

Our results demonstrate that *CDH3 *gene expression may increase in Riedel's thyroiditis, as well. Cadherins, such as *CDH3*, are integral membrane glycoproteins responsible for calcium-dependent cell-cell adhesion. Our contemporary knowledge about the role of P-cadherin in fibrosis and autoimmune processes is scanty and, so far, it has been only demonstrated that aberrant expression of E-cadherin/β-catenin complex is associated with a wide variety of human malignancies and fibrosis [13].

The association between increased expression levels of *PIK3CA *and *CDH3 *genes and Riedel's thyroiditis is not well-defined. However, the increased expression of *PIK3CA *and *CDH3 *genes in our case report and in previous studies of other authors on various malignancies may suggest possible molecular relation between Riedel's thyroiditis and certain neoplastic processes, the relation which requires further genetic evaluation.

Low expression levels of the remaining studied genes could be associated with destruction of glandular thyroid tissue. The levels of *TGFB1, COL1 *and *CDKN1C *genes expression were comparable in Riedel's thyroiditis and in macroscopically unchanged tissue. It is to be emphasized that high expression levels of *TGFB1 *and of *COL1A1 *genes in chronic thyroiditis and its correlation with thyroid fibrosis has previously been observed [14]. These results call into question the "intrathyroidal hypothesis" of Riedel's disease pathogenesis. One should remind once again that gene expression studies in Riedel's thyroiditis are difficult to perform, mainly due to fibrosis, resulting in scarce thyroid specimens and - in consequence - small amount of genetic material.

## Consent

Written informed consent was obtained from the patient for publication of this report and any accompanying images.

## Competing interests

The authors declare that they have no competing interests.

## Authors' contributions

KW-D designed and coordinated the study, carried out the molecular genetic studies, writing a manuscript. AD, SS and JS participated in coordination of the study. AL senior supervision, writing a manuscript. All authors have read and approved the final manuscript.
